# Editorial: Sports, nutrition and public health: analyzing their interconnected impacts

**DOI:** 10.3389/fpubh.2026.1861120

**Published:** 2026-06-02

**Authors:** Tianyuan He, Jian Sun, Minghui Li

**Affiliations:** 1 School of Athletic Training, Guangzhou Sport University, Guangzhou, China; 2 Key Laboratory of Human-Computer Intelligent Interaction for Athletic Performance and Health Promotion, Guangzhou Sport University, Guangzhou, Guangdong, China; 3 Department of Clinical Pharmacy and Translational Science, University of Tennessee Health Science Center, Memphis, TN, United States

**Keywords:** diet, nutrition, physical activity (exercise), public health, sports

## Introduction

Regular physical activity is not only an important means of maintaining individual physical and mental health but also a vital intervention within the global public health system for the prevention and management of cardiovascular diseases, type 2 diabetes, and various chronic conditions. Research indicates that long-term adherence to physical activity yields significant cumulative benefits in preventing major chronic diseases (1). Furthermore, the protective effect of exercise in reducing all-cause mortality spans every stage of adult life (2).

However, the effectiveness of physical activity depends largely on the scientific support of dietary nutrition. Proper nutritional intake, as the material foundation for metabolic homeostasis and tissue repair, has a decisive impact on preventing non-communicable diseases. Current evidence suggests that both improving the quality of overall dietary patterns and exploring more targeted precision nutrition interventions are central to maintaining metabolic balance and long-term health (3). As highlighted by Lv et al., direct comparisons of various dietary patterns further clarify their specific advantages in managing metabolic syndrome.

Based on this, integrating physical activity with dietary nutrition has become pivotal in addressing global public health challenges. A close physiological link exists between exercise and nutrition; their synergy can more effectively improve metabolic health and prevent disease than any single intervention (4, 5). Therefore, constructing comprehensive intervention programs that prioritize both exercise and nutrition, and integrating them into public health systems, has become an urgent priority (6).

The importance of these integrative strategies is increasingly evident given the escalating global burden of disease. Projections from global burden of disease studies indicate a substantial increase in the global diabetes population by 2050, a significant portion of which is directly linked to overweight and obesity (7). Faced with this challenge, the public health sector has an imperative need for systemic intervention models. This Research Topic aims to synthesize global insights in the field. Through the systematic integration of 55 interdisciplinary research contributions, we have constructed a cohesive logical framework: from understanding macro-social contexts and environmental barriers to analyzing micro-mechanisms of lifestyle interactions, developing precision assessment tools and evidence synthesis databases, and finally achieving inclusive health translation for diverse populations.

## Macro-perspectives and social determinants

A core contribution of this Research Topic is that it moves beyond a focus on disease alone to examine the environmental factors underlying global health challenges from a macro perspective. By integrating multiple studies based on Global Burden of Disease (GBD) data, this Research Topic presents the severe challenges currently facing public health: the burden of endocrine, metabolic, and immune system diseases will continue to intensify over the coming decades, with highly unequal global distributions, as shown by Liang J. et al. and Pan et al.. Research further reveals that obesity, as a central risk factor, not only exacerbates individual metabolic dysfunction but also significantly increases the risk of chronic conditions such as knee osteoarthritis, as discussed by Xu J. et al. These epidemiological findings collectively underscore that the prevention and management of chronic diseases must be elevated to the level of global policy-making, a view emphasized in the expert consensus roadmap by Wirnitzer et al..

Concurrently, this Research Topic provides a deep analysis of how social determinants profoundly influence individual health trajectories. Evidence from Li M. et al. demonstrates that socioeconomic status often acts as a key mediator of physical fitness by constraining resource access and the development of motor skills. Furthermore, this Research Topic highlights a modern challenge in the digital era, with Li H. et al. noting that digital literacy has emerged as a new impediment to public engagement in fitness activities. Imbalances in social resource allocation—such as the mismatch between supply and demand for sports services in rural areas (Zhang, He, et al.) and the difficulties in accessing nutritious food faced by female athletes in specific campus environments (Uriegas et al.)—further highlight the urgent need to build supportive health environments. These environmental influences take root in early life, where family background and the accessibility of sports facilities are critical factors in determining whether children and adolescents develop regular exercise habits, as explored by Qin et al. and Sheng et al.. Having identified these macro-contexts that shape health behaviors, it is necessary to further explore how individual lifestyle choices directly interact with physiological metabolism under these complex social forces.

## Lifestyle, metabolic health, and chronic disease prevention

Having understood the impact of the macro-environment, this Research Topic utilizes a series of studies to analyze the complex interaction mechanisms among various lifestyle elements. The research here demonstrates that health status is a system of deeply interconnected behaviors. Regarding risk, Wang A. et al. quantified the synergistic amplification of systemic inflammation caused by the accumulation of adverse behaviors and metabolic risk factors, while Liu Z. et al. explored the specific associations between domain-specific physical activity, sedentary behavior, and the risk of binge drinking. Conversely, this Research Topic provides evidence for the critical role of adhering to a healthy lifestyle in counteracting the risks of pre-metabolic syndrome, as discussed by Xu Y. et al., and highlights the potential regulatory value of specific nutrients, such as selenium, in managing diabetes risk (Alfawaz et al.).

Another highlight of this Research Topic is its revelation of how different health behaviors drive or constrain one another. For example, Zhao and Huang show that physical exercise can significantly inhibit smoking behavior, while Huang W. et al. demonstrate how active exercise can optimize dietary patterns by mediating negative emotions. Furthermore, the research emphasizes the need for refined lifestyle management; Sung E. et al. analyzed the interactions between sleep, diet, and exercise in preventing metabolic syndrome, while Zheng et al. examined the combined impact of pro-inflammatory diets and physical activity on frailty in the older adults. Mechanistic explorations also extend to broader conditions, including the complex associations between physical activity and the risk of chronic obstructive pulmonary disease (Kou et al.) and kidney stones (Wang Y. et al.). Understanding these complex lifestyle interactions leads to a central theme: maintaining metabolic health must shift from single-dimensional interventions toward a holistic medical perspective that integrates exercise, nutrition, sleep, and mental health.

## Seeking precision and scientific intervention strategies

The effectiveness of scientific interventions must be built upon reliable, evidence-based data. This Research Topic has constructed a comprehensive “evidence database” by aggregating high-level synthesis studies. In the field of exercise intervention, research has confirmed the high efficiency of sprint interval training (SIT), as shown by Liang W. et al., refined the clinical parameters for isometric training in blood pressure regulation (Yan et al.), and validated the immediate value of “exercise snacks” and varied periodized training for improving metabolism and performance, as demonstrated by Chang et al. and Zhang, Ya et al.. In terms of nutrition and rehabilitation, evidence synthesis by Liu S. et al. affirms the potential of probiotics in weight management, while Zhang, Zhang et al. clarify the multiple benefits of physical exercise as an adjuvant therapy for cancer. Furthermore, Zhu Y. et al. reveal the bidirectional link between depression and sarcopenia. Additionally, network meta-analyses on the effects of dietary supplements by Deng et al. and Zhao R., et al., alongside specialized reviews by Liao et al. and Zhao J. et al., provide a robust scientific basis for precision interventions.

Simultaneously, this Research Topic pays particular attention to innovations in assessment metrics and methodologies, which are the technical prerequisites for precision health management. A series of studies on novel biomarkers and body composition metrics—including the UHR ratio (Wang F. et al.; Wang Y. et al.), TyG-related indices (Zhang, Wang et al.), the FFM/FM ratio (Sung J. et al.), and the WWI index (Zhang, Tang et al.)—have validated their value in identifying risks for diabetes, obesity, and cardiovascular issues. By establishing body fat percentage percentile curves for Chinese adults (Tu et al.) and optimizing skeletal muscle mass predictive equations for specific athletes (Ojeda-Aravena et al.), this Research Topic provides precise assessment standards for diverse populations. More prospectively, the application of machine learning models in risk prediction by Zhang, Peng et al. and Zhao R. et al., as well as the development of omics-driven precision nutrition frameworks proposed by Penggalih et al. and Zhu Z. et al., demonstrate the field's advancement toward intelligent health management. Relying on this robust methodological support, it becomes possible to implement cutting-edge intervention protocols across the lifespan for diverse populations.

## Precision health management for diverse populations

The ultimate value of scientific discovery lies in its translation into real-world applications. The research in this Research Topic fully embodies the person-centered philosophy of public health, emphasizing that intervention strategies must account for an individual's specific stage in the life cycle. In childhood and adolescence, Li Y. et al. explored the profound impact of dietary behaviors and BMI levels on physical development. Research focused on specific life stages and professional groups, such as the investigation by Szablewska et al. on the impact of cesarean sections on later maternal activity, the assessment of food safety awareness among pregnant women by Varol and Aksoy Kendilci, and the analysis of health behavior determinants among firefighters by Dudziński et al., provides essential support for filling existing gaps in health services for targeted populations.

Furthermore, this Research Topic demonstrates how to customize precise intervention systems based on specific physiological risks and professional demands. Empirical studies by Chouk et al. and Yamazaki et al. on patients with type 2 diabetes, alongside research by Allam et al. and Wang Z. et al. on the older adults, demonstrate the significant synergistic advantages of “exercise + nutrition” combined protocols and physical aids. The Research Topic also examines nutritional attitudes among athletes, as detailed by Huang T. et al. and Sahin et al., and provides rigorous assessments of adjuvant therapies in cancer recovery, as shown by Celorrio San Miguel et al., highlighting the necessity of tailoring support systems to specific requirements. In summary, these studies across diverse groups collectively argue that successful public health interventions must shift from “generalized recommendations” to “personalized applications” based on physiological, occupational, and environmental characteristics.

## Conclusion and outlook

The 55 research contributions gathered in this Research Topic collectively delineate how exercise and nutrition are deeply interwoven to reshape modern public health. From the analysis of macro-social determinants and micro-physiological interactions to the enhancement of evidence-based databases and innovations in precision assessment tools, these findings not only deepen our understanding of the complex “exercise-nutrition-health” system but also provide scientific guidance for developing inclusive, efficient, and targeted public health strategies. Through empirical research across diverse populations, this Research Topic effectively demonstrates that the success of modern public health depends not only on changes in individual behavior but also on the improvement of social environments and the advancement of precision intervention technologies.

Looking ahead, to fully release the potential of exercise and nutrition science in enhancing healthy lifespan for all, the field must continue to focus on the following areas:

- **Strengthening precision intervention and digital empowerment:** Actively embrace artificial intelligence, machine learning, and omics technologies to drive the transition of health management from “generalized recommendations” to “precision customization” based on individual molecular responses and behavioral patterns.
- **Promoting systematic interdisciplinary integration:** Use policy guidance and mechanism innovation to deeply integrate evidence-based exercise and nutrition prescriptions into primary healthcare systems and community public services, building a systematic health promotion model that covers the entire life cycle.
- **Ensuring health equity and inclusive development:** Future research and practice should prioritize resource-limited areas, vulnerable groups, and individuals at specific physiological stages. By addressing social determinants, we must ensure that scientific health interventions benefit every individual equitably.

We hope this Research Topic stimulates broader interdisciplinary collaboration and deep reflection, collectively driving the findings of exercise and nutrition science more accurately and extensively into public health policy and clinical practice, ultimately making a substantial contribution to a more vibrant, healthier, and resilient future society.
